# *APOE* and *KLF14* genetic variants are sex-specific for low high-density lipoprotein cholesterol identified by a genome-wide association study

**DOI:** 10.1590/1678-4685-GMB-2021-0280

**Published:** 2022-02-21

**Authors:** Ying-Hui Lee, Ya-Sian Chang, Chih-Chang Hsieh, Rong-Tsorng Wang, Jan-Gowth Chang, Chung-Jen Chen, Shun-Jen Chang

**Affiliations:** 1 Kaohsiung Veterans General Hospital, Department of Pathology and Laboratory Medicine, Division of Microbiology, Kaohsiung, Taiwan; 2 China Medical University Hospital, Center for Precision Medicine, Taichung, Taiwan; 3 Kaohsiung Medical University, Office of Library and Information Services, Kaohsiung, Taiwan; 4 Tunghai University, Department of Statistics, Taichung, Taiwan; 5 Kaohsiung Medical University Hospital, Department of Internal Medicine, Division of Rheumatology, Kaohsiung, Taiwan; 6 National University of Kaohsiung, Department of Kinesiology, Health and Leisure Studies, Kaohsiung, Taiwan

**Keywords:** Genome-wide association study, High-density lipoprotein cholesterol, sex-specific, KLF14, APOE

## Abstract

To demonstrate the loci that relate to high-density lipoprotein cholesterol (HDL-C) levels and genetic sex heterogeneity, we enrolled 41,526 participants aged between 30 and 70 years old from the Taiwan Biobank in a genome-wide association study. We applied the Manhattan plot to display the *p-*values estimated for the relationships between loci and low HDL-C. A total of 160 variants were significantly associated with low HDL-C. The genotype TT of rs1364422 located in the *KLF14* gene has 1.30 (95% CI=1.20 - 1.42) times the risk for low-HDL compared to genotype CC in females (log(-p) =8.98). Moreover, the genes *APOC1*, *APOE*, *PVRL2*, and *TOMM40* were associated significantly with low-HDL-C in males only. Excluding the variants with high linkage disequilibrium, we revealed the rs429358 located in *APOE* as the major genetic variant for lowering HDL-C, in which genotype CT has 1.24 (95% CI= 1.16 - 1.32) times the risk. In addition, we also examine 12 genes related to HDL-C in both sexes, including *LPL*, *ABCA1*, *APOA5*, *BUD13*, *ZPR1*, *ALDH1A2*, *LIPC*, *CETP*, *HERPUD1*, *LIPG*, *ANGPTL8,* and *DOCK6*. In conclusion, low-HDL-C is a genetic and sex-specific phenotype, and we discovered that the *APOE* and *KLF14* are specific to low-HDL-C for men and women, respectively.

## Introduction

The level of low high-density lipoproteins cholesterol (HDL-C) is a common indicator of metabolic syndrome and dyslipidemia (Lazo-Porras et al., 2016). Low HDL-C is also a typical component of familial combined hyperlipidemia (FCHL) (de Graaf and Stalenhoef, 1998; Soro et al., 2003) in which the risk for premature coronary artery disease (CAD) is 2- to 10-fold greater than in the general population (Goldstein et al., 1973; Genest *et al.*, 1992; Hopkins et al., 2003). HDL-C also showed a strong inherited basis with heritability estimates of 40-60% (Weissglas-Volkov and Pajukanta, 2010). Inversely, the higher HDL-C levels showed a protective effect from CAD even after adjustment of non-HDL-C and triglycerides (Emerging Risk Factors Collaboration et al., 2009). Given the public health relevance and the strong genetic component (von Eckardstein et al., 2000; Weiss *et al.*, 2006; Emerging Risk Factors Collaboration *et al.*, 2009; Laks et al., 2011), considerable efforts have been made to elucidate the genetic architecture for lower HDL-C levels. In the past, several large-scale studies, culminating in the 2013 Global Lipids Genetics Consortium, have contributed to the discovery and validation of genetic loci associated with HDL-C levels (Teslovich et al., 2010; Willer et al., 2013). Interestingly, the disconcordant heritability obtained from different populations and distinct sexes from previous HDL studies (Weiss *et al.*, 2006; Souren et al., 2007) indicated that genetic factors of HDL-C may act in a population- and/or sex-specific manner. 

Previously, most genome-wide association studies (GWAS) were carried out in Caucasian populations. Correspondingly, there was a dearth of genetic studies investigating HDL-C in non-European populations, especially in different sexes. While most associated loci relate to the genetic architecture of HDL-C levels in populations worldwide, the degree and specific types of this association are not always consistent. For example, heterogeneity of lower HDL-C effects between males and females suggested that HDL-C levels might be regulated differentially across sexes (Teslovich et al., 2010). A close evaluation of these details is crucial in setting the right public health policies so that the economic and health burden can be reduced. This was evidenced by increases in the trait variance after adding population-specific signals (Wu et al., 2013). Therefore, ethnicity- and sex-specific study may be necessary for understanding population-specific HDL-C genetic architecture and resource planning.

This study presents genetic findings in a Taiwanese cohort of both sexes from the Taiwan Biobank (TWB). Identifying genetic loci related to variation in the phenotype may help us to understand how lipid metabolism functions and how effective strategies can be developed to prevent and treat lower HDL-C according to different sexes.

## Subjects and Methods

### Study population

We aimed to explore the genetic variants associated with HDL-C dysfunction and enrolled 41,526 participants aged between 30 and 70 years old from TWB, and we matched them by sex and age. TWB, a nationwide database for research, combines genomic profiles with lifestyle patterns from people in Taiwan to explore the relationships among genetics, the environment, and the etiology/progression of diseases. Each participant underwent biochemical testing (with blood samples) and a physical examination. Blood HDL-C levels were measured after more than 8 hours of fasting. HDL-C levels of the study population were grouped as a new dichotomy variable low-HDL-C for men who have HDL-C <=40 mg/dl, or women who have HDL-C <=50 mg/dl; others were defined as normal HDL-C. The current uses of tobacco smoking and alcohol consumption were applied for the confounding analysis of genetic variants relating to low-HDL-C. The ethics committee of the China Medical University Hospital Institutional Review Board in Taiwan (CMUH108-REC1-091) has approved this project. Both the Declaration of Helsinki and the Good Clinical Practice Guidelines were followed and informed consent granted by all participants. Both the Declaration of Helsinki and the Good Clinical Practice Guidelines have observed, and informed consent have been granted by all participants.

### Genotyping and quality control

All samples were genotyped using Affymetrix Axiom genotyping array (chip: TWB2), including 680K SNPs. Quality control (QC) was applied to leave out those SNPs with low call rate (< 95%), minor allele frequencies (MAF) less than 0.05, and deviations from the Hardy-Weinberg equilibrium (p < 0.05; library HardyWeinberg of R program). 

### Statistical analysis

We used a total of 266,556 SNPs that passed the quality control for GWAS. The clinical characteristics between subjects with and without low-HDL-C were compared, applying a chi-square test for categorical variables and a t-test for continuous variables. The *p-*values estimated by the chi-square test (chisq.test function of R program) for the association between genetic variants located on each chromosome pair (22 autosomes and X, Y sex chromosomes) and low- HDL-C were presented in Manhattan plots (library qqman), using the R-program provided by Turner (Turner, 2014). We applied a chi-square test for trend to assess the dose-response effect of various number of alternative allele (or genotypes) on the low-HDL-C rate. We conducted a logistic regression model for estimating the odds ratios (ORs) as well as 95% confidence intervals (95% CI) that followed Poisson distribution for the associations between genetic variants and low-HDL-C after adjustment of variables including total cholesterol, triglycerides, and body mass index (BMI) (library aod of R program). Moreover, an analysis of variance (ANOVA) was used to detect the mean differences of HDL-C level among genotypes. The Locuszoom plot was employed to visualize the regional strength and associations between loci and low-HDL-C related to local linkage-disequilibrium (LD), recombination patterns, and genomic position (locuszoom v1.4 for python v2.7) (Pruim et al., 2010). For the GWAS, after application of the Bonferroni correction for multiple testing, the significance was determined at *p* < 1 × 10^−8^. For variables such as BMI, total cholesterol, and triglycerides, the significance was determined at *p*< 0.05. We used PLINK (v1.9), PERL (v5.16) and R (v3.6) programs to mine raw data, estimate *p-*values and draw plots in CentOS platform (v7.0). 

## Results

A total of 20,763 male participants and the same number of female participants from TWB were included in the study. Each male was matched by one female with the same age to ensure the mean age did not significantly differ between genders (mean age=50.71 years old, SD=11.32, *p*=1.000). Considering the associations between demographic characteristics and low-HDL-C, the results showed that diastolic blood pressure, triglycerides, systolic blood pressure, waist circumference, BMI, and fasting glucose were significantly associated with low HDL-C in both males and females (all *p-*values <0.001; Table 1).

**Table 1 -:** Relationship between low-HDL-C and basic demographic and biochemical markers.

	Males (n=20,763)			Females (n=20,763)		
	HDL-C Lower	HDL-C normal	*p-*values	HDL-C Lower	HDL-C normal	*p-*values
Age (mean ±SD))	50.7±11.1	50.8±11.3	0.804	51.2±11.2	50.6±11.3	0.0001
BMI (mean ±SD)	26.8±3.6	24.8±3.3	<0.001	25.2±3.9	22.9±3.4	<0.001
WC (mean ±SD)	91.9±9.3	86.7±9.0	<0.001	84.9±9.7	79.1±9.2	<0.001
SBP (mean ±SD)	128.9±17.3	126.9±17.5	<0.001	121.2±18.9	116.7±18.7	<0.001
DBP (mean ±SD)	80.1±11.1	78.5±10.9	<0.001	73.0±10.5	70.5±10.5	<0.001
Fasting glucose (mean ±SD)	103.3±29.2	98.2±21.5	<0.001	98.4±25.0	92.3±15.7	<0.001
Triglycerides (mean ±SD)	207.0±195.5	115.6±77.4	<0.001	145.7±96.6	84.1±43.5	<0.001

HDL-C: high-density lipoprotein cholesterol; BMI: body mass index; WC: waist circumference; SBP: systolic blood pressure; DBP: diastolic blood pressure

**Figure 1 -:**
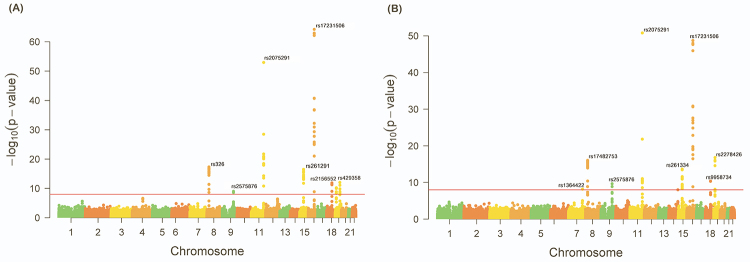
The Manhattan plots showing the calculated *p-*values for SNPs in all 23 chromosome pairs for the associations with low high-density lipoprotein cholesterol in men (A) and in women (B). The horizontal line indicates the cut-off value for *p-*values (1 × 10-8). The *p-*values were estimated by chi-square test.

**Figure 2 -:**
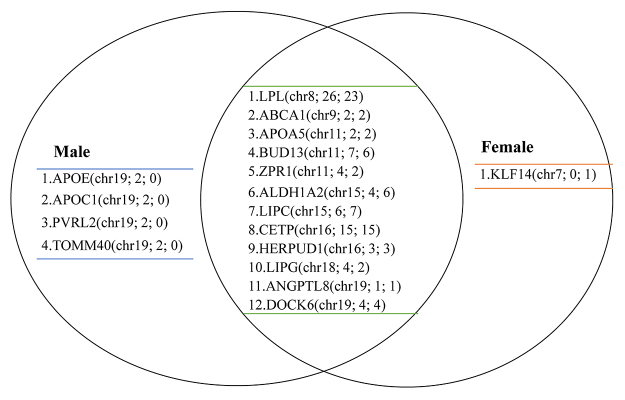
Summary chart of all the loci is significantly related to low high-density lipoprotein cholesterol (HDL-C). And the gene name of the significant variants located was noted. The chromosome number and number of loci significantly related to HDL-C in men and women were included in the parenthesis. The left part indicates those genes related significantly to low-HDL-C were found only in men, such as *APOE*, *APOC1*, *PVRL2*, and *TOMM40*. The right part indicates that the polymorphism rs1364422 in gene *KLF14* was significantly related to women, but the same effect was not found in men. The middle part indicates the genes were related to low-HDL-C found in both sexes.

The Manhattan plot showed all *p-*values of loci related to low-HDL-C across 23 pairs of chromosomes and showed a total of 160 variants significantly associated with low HDL-C located on chromosomes 7, 8, 9, 11, 15, 16, 18 and 19 (Figure 1). In addition, all the variants significantly related to low-HDL-C were grouped by sex into their belonging gene (or near gene), the detailed information is listed in Table S1 for males and Table S2 for females. Notably, more variants in chromosome 19 of males showed significant association with low-HDL-C, in comparison to the total number of loci in females (Figure 1A). Chromosome 7 revealed that the female-specific variant, rs1364422, located in the *KLF14* gene, was significantly associated with low-HDL-C (Figure 1B). The variants in genes *APOC1*, *APOE*, *PVRL2*, and *TOMM40* were significant only in males; moreover, the variants in genes *LPL*, *ABCA1*, *APOA5*, *BUD13*, *ZPR1*, *ALDH1A2*, *LIPC*, *CETP*, *HERPUD1*, *LIPG*, *ANGPTL8*, and *DOCK6* have shown significant results in both genders (Figure 2).

**Figure 3 -:**
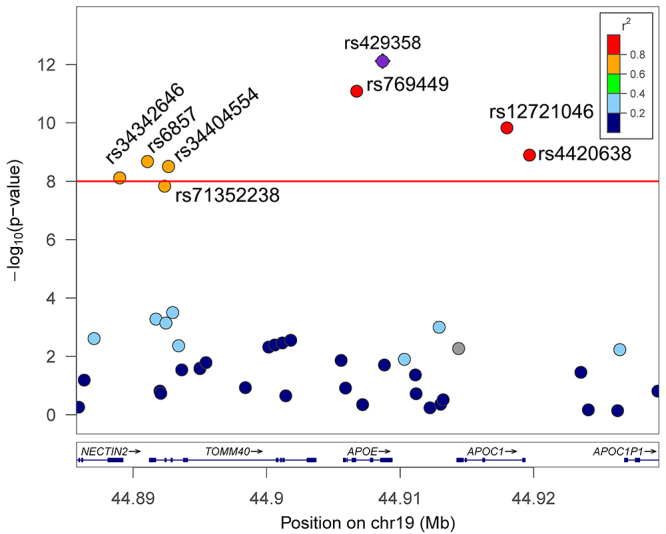
Locuszoom plot illustrates the linkage disequilibrium and recombination rate between four genes in chromosome 19 in male participants. The genes include *APOE*, *APOC1*, *PVRL2*, and *TOMM40*. It showed the polymorphism rs429358 was the major variant for low high-density lipoprotein cholesterol in men.

Concerning the LD between loci that significantly related to low-HDL-C located in chromosome 19 in males, the Locuszoom plot highlighted recombination rates that existed within these loci (r^2^ >0.5). The loci consist of rs429358 (*APOE*), rs34342646 (*PVRL2*), rs6857 (*PVRL2*), rs34404554 (*TOMM40*), rs71352238 (*TOMM40*), rs769449 (*APOE*), and rs4420638 (*APOC1*) (Figure 3). The high LD degree of these significant variants suggests that the rs429358 in the *APOE* gene is the only major variant for relating to lower HDL-C compared to the other variants located in genes *PVRL2*, *APOC1*, and *TOMM40*.

Compared with those with genotype CC of rs1364422 located in the *KLF14* gene, cases with genotype TT had 1.30 (95% CI=1.20 - 1.42) times the risk for low-HDL-C in females (-log(p)=8.98; Table 2), but the same effect was not indicated in males (*p*=0.166). Furthermore, those with genotype TT also have a higher prevalence of low-HDL-C than those with genotypes CT or CC in females (34.52%, 31.27%, 28.79%, respectively; *p* for trend <1 x 10^-8^). The above result suggests that the rs1364422 T allele could be different from the C allele in physiologic function. For the male-specific genes related to low-HDL-C, we only demonstrated the OR and 95% CI of the top significant variant of each gene in Table 2. The polymorphism rs429358 located in the *APOE* gene acted as the major candidate variant for the male-specific variant through the LD exclusion by Locuszoom plot. The result showed that those with genotype CT of rs429358 had 1.24 (95% CI= 1.16 - 1.32) times the risk of becoming lower HDL-C, which occurred only in males (-log(p)=9.22). The other polymorphisms located in genes *APOC1*, *PVRL2*, and *TOMM40* also showed high associations with lower HDL-C; the *p-*values have fallen in the range 1 x10^-6^ and 1 x10^-8^ in the male group (Table 2).

**Table 2 -:** ORs and allele information of polymorphisms which were sex-specific related to low-HDL-C.

SNP	Chr.	Position	Gene	Alt.	Males (n=20763)			Females (n=20763)		
					Case (%)	OR (95% CI)	-log (*p-*value)	Case (%)	OR (95% CI)	-log (*p-*value)
rs1364422	7	130761222	*KLF14*	T						
		TT			973 (25.79)	0.94(0.86-1.03)	0.81	1282 (34.52)	1.30(1.20-1.42)	8.98
		CT			2755 (27.39)	1.02(0.95-1.09)	0.20	3153 (31.27)	1.13(1.05-1.20)	3.27
		CC			1867 (27.06)	1.0		1996 (28.79)	1.0	
rs429358	19	44908684	*APOE*	C						
		CC			42 (34.15)	1.32(0.96-1.76)	1.11	47 (38.84)	1.28(0.94-1.68)	1.02
		CT			1032 (32.14)	1.24(1.16-1.32)	9.22	1109 (34.39)	1.13(1.06-1.20)	3.62
		TT			4453 (25.95)	1.0		5103 (30.45)	1.0	
rs4420638	19	44919689	*APOC1*	G						
		GG			69 (33.82)	1.26(0.99-1.58)	1.30	69 (34.33)	1.13(0.88-1.42)	0.49
		GA			1159 (30.86)	1.18(1.11-1.26)	6.42	1274 (33.56)	1.10(1.04-1.17)	2.74
		AA			4374 (26.04)	1.0		5102 (30.44)	1.0	
rs34342646	19	44884873	*PVRL2*	A						
		AA			49 (36.03)	1.38(1.03-1.80)	1.59	47 (36.15)	1.18(0.87-1.56)	0.60
		GA			964 (31.27)	1.20(1.11-1.28)	6.32	1048 (33.44)	1.09(1.02-1.17)	2.09
		GG			4589 (26.16)	1.0		5348 (30.58)	1.0	
rs34404554	19	44891079	*TOMM40*	G						
		GG			50 (35.71)	1.37(1.02-1.78)	1.54	47 (35.07)	1.15(0.85-1.51)	0.46
		CG			965 (31.19)	1.19(1.11-1.28)	6.16	1054 (33.47)	1.09(1.02-1.17)	2.14
		CC			4584 (26.16)	1.0		5341 (30.57)	1.0	

OR: odds ratio; 95% CI: 95% confident intervals; The ORs were estimated by logistic regression after adjustment of body mass index, total cholesterol and triglycerides. HDL-C: high-density lipoprotein cholesterol; Chr: chromosome; Alt: alternative allele.

We estimated the mean differences of HDL-C among genotypes of the sex-specific polymorphisms related to low-HDL-C, such as rs1364422, rs429358, rs4420638, rs34342646 and rs34404554. Except for the polymorphism rs1364422, which did not reveal any significant difference of HDL-C related to the genotypes in males (*p*=0.298; Table 3), the mean difference in females also remains high significant difference (-log(p)=4.76). Besides, the other variants showed highly significant differences of the HDL-C with different genotypes in both sexes (all *p-*values < 0.0001).

**Table 3 -:** Mean values of HDL-C among the polymorphisms which were sex-specific related to low-HDL-C.

SNP	Genotypes	Males (n=20763)			Females (n=20763)		
		No.	mean ±SD (mg/dl)	-log(p) post hoc	No.	mean ±SD (mg/dl)	-log(p) post hoc
rs1364422							
	TT^a^	3773	48.04 ±11.22	0.526	3714	57.11 ±13.25	4.76 a<c, b<c
	CT^b^	10059	47.76 ±11.09		10082	57.94 ±13.26	
	CC^c^	6900	47.71 ±11.09		6933	58.37 ±12.97	
rs429358							
	CC^a^	123	46.43 ±10.98	10.89 b<c	121	55.15 ±13.25	9.63 b/c, a<c
	CT^b^	3211	46.55 ±11.03		3225	56.60 ±13.06	
	TT^c^	17158	48.04 ±11.12		16761	58.19 ±13.20	
rs4420638							
	GG^a^	204	46.22 ±11.29	9.80 b<c	201	56.39 ±12.51	7.38 b<c
	GA^b^	3756	46.76 ±10.87		3796	56.87 ±13.03	
	AA^c^	16795	48.04 ±11.15		16761	58.19 ±13.20	
rs34342646							
	AA^a^	136	45.76 ±11.17	7.89 b<c	130	55.59 ±13.01	6.31 b<c
	GA^b^	3083	46.77 ±11.03		3134	56.86 ±12.94	
	GG^c^	17543	47.99 ±11.12		17491	58.14 ±13.20	
rs34404554							
	GG^a^	140	45.79 ±11.06	7.74 b<c	134	55.87 ±13.04	6.36 b<c
	CG^b^	3094	46.78 ±11.04		3149	56.84 ±12.92	
	CC^c^	17521	47.99 ±11.12		17469	58.14 ±13.21	

The ORs of loci and the allele information which significantly related to low-HDL-C occurred in both genders are listed in Table 4. We only listed the top significant variant of each gene related to low-HDL-C. The results revealed that the variants with homozygous genotypes of polymorphisms rs261291 (*ALDH1A2*), rs2070895 (*LIPC*), rs17231506 (*CETP*), rs247616 (*HERPUD1*), and rs9958734 (*LIPG*) have strong protective effects against lowering HDL-C compared to those with wild type (all -log(p) >10). One exception is that a variant with heterozygous genotype of polymorphism rs17482753 (*LPL*) also showed its protective effect in both genders (-log(p) >=10). Moreover, the loci with homozygous genotypes of polymorphisms Affx4282911 (*APOA5*), rs7350481 (*BUD13*), rs2160669 (*ZPR1*), rs2278426 (*ANGPTL8*), and rs3760782 (*DOCK6*) showed a deleterious effect on HDL-C (all -log(p) >6); especially, all the polymorphisms showed a higher-dose response of lowering HDL-C levels with the number of allelic variants (*p-*values test for trend, all -log(p) >10 in both genders). 

**Table 4 -:** The ORs and allele information of polymorphisms which were significantly related to low-HDL-C in both males and females.

Variants	Chr.	Position	Gene	Alt.	Males (n=20763)			Females (n=20763)		
					Case (%)	OR (95% CI)	-log (*p-*value)	Case (%)	OR (95% CI)	-log (*p-*value)
rs17482753	8	199751350	*LPL*	T						
		TT			35 (18.13)	0.64(0.45-0.88)	2.03	47 (21.08)	0.65(0.48-0.86)	2.47
		GT			822 (22.03)	0.78(0.72-0.84)	10.23	995 (25.80)	0.80(0.74-0.85)	10.35
		GG			4740 (28.20)	1.0		5396 (32.39)	1.0	
Affx4282911	11	116790676	*APOA5*	A						
		AA			71 (66.98)	2.65(2.07-3.32)	15.38	64 (66.67)	2.28(1.76-2.89)	10.24
		CA			952 (37.06)	1.46(1.36-1.57)	25.96	1087 (42.21)	1.44(1.35-1.54)	27.45
		CC			4575 (25.32)	1.0		5290 (29.26)	1.0	
rs7350481	11	116715567	*BUD13*	C						
		CC			457 (38.12)	1.55(1.41-1.71)	17.78	496 (40.82)	1.42(1.29-1.56)	12.59
		TC			2185 (29.20)	1.19(1.13-1.26)	9.22	2481 (33.20)	1.16(1.10-1.22)	7.40
		TT			2958 (24.52)	1.0		3469 (28.74)	1.0	
rs2160669	11	116776891	*ZPR1*	T						
		TT			354 (38.23)	1.52(1.36-1.70)	13.32	341 (39.84)	1.34(1.20-1.49)	6.62
		CT			1952 (29.20)	1.16(1.10-1.23)	6.98	2184 (32.43)	1.09(1.03-1.15)	2.89
		CC			3285 (25.08)	1.0		3905 (29.76)	1.0	
rs261291	15	58387979	*ALDH1A2*	C						
		CC			759 (21.56)	0.66(0.60-0.72)	17.56	926 (26.75)	0.72(0.66-0.79)	11.67
		TC			2731 (27.10)	0.89(0.83-0.95)	3.32	3125 (30.77)	0.88(0.83-0.94)	3.82
		TT			2104 (37.61)	1.0		2375 (33.50)	1.0	
rs2070895	15	58431740	*LIPC*	A						
		AA			700 (23.33)	0.71(0.64-0.78)	11.51	831 (26.76)	0.72(0.66-0.79)	11.34
		GA			2518 (25.64)	0.80(0.75-0.86)	10.17	2937 (30.33)	0.86(0.81-0.92)	5.38
		GG			2374 (30.05)	1.0		2663 (33.58)	1.0	
rs17231506	16	56960616	*CETP*	T						
		TT			71 (12.46)	0.41(0.32-0.51)	13.06	93 (15.71)	0.46(0.37-0.56)	12.85
		CT			1128 (19.80)	0.65(0.61-0.70)	37.04	1419 (24.97)	0.73(0.69-0.78)	24.17
		CC			4391 (30.40)	1.0		4923 (34.06)	1.0	
rs247616	16	56955678	*HERPUD1*	T						
		TT			67 (11.92)	0.39(0.31-0.50)	13.47	92 (15.54)	0.46(0.37-0.56)	13.00
		CT			1129 (19.92)	0.66(0.62-0.70)	35.58	1418 (25.08)	0.74(0.70-0.78)	23.22
		CC			4402 (30.31)	1.0		4930 (34.00)	1.0	
rs9958734	18	49592028	*LIPG*	C						
		CC			792 (23.12)	0.72(0.65-0.79)	11.43	911 (27.02)	0.73(0.67-0.80)	10.79
		TC			2634 (26.44)	0.86(0.80-0.92)	5.18	3066 (30.56)	0.87(0.82-0.93)	4.47
		TT			2163 (29.55)	1.0		2451 (33.53)	1.05	
rs2278426	19	11239812	*ANGPTL8*	T						
		TT			438 (32.74)	1.29(1.17-1.43)	6.27	557 (39.70)	1.37(1.25-1.50)	11.11
		CT			2257 (28.35)	1.12(1.06-1.18)	4.25	2580 (32.36)	1.12(1.06-1.17)	4.48
		CC			2887 (25.32)	1.0		3285 (29.02)	1.0	
rs3760782	19	11235874	*DOCK6*	T						
		TT			451 (32.87)	1.30(1.17-1.43)	6.50	562 (39.03)	1.35(1.23-1.47)	10.17
		CT			2254 (28.28)	1.11(1.05-1.18)	3.95	2586 (32.54)	1.12(1.07-1.18)	5.00
		CC			2889 (25.37)	1.0		3287 (28.97)	1.0	

OR: odds ratio; 95% CI: 95% confidence intervals; The ORs were estimated by logistic regression after adjustment of body mass index, total cholesterol and triglycerides. HDL-C: high-density lipoprotein cholesterol; Chr: chromosome; Alt: alternative allele. OR equal to 1.0 indicates that acts as referent group.

Moreover, we further estimated the relationships between low-HDL-C and current uses of tobacco smoking and alcohol consumption. The results showed that both effects on low-HDL-C were dominant in males, that tobacco smoking exhibited a higher risk of causing low-HDL-C (OR=1.87, 95% CI=1.74-2.01, -log(p) =65.61), and alcohol consumption showed a protective effect (OR=0.70, 95% CI =0.64-0.77, -log(p) =12.19; Table 5). To avoid the confounding effects of tobacco smoking and alcohol consumption on low-HDL-C, we further estimated the associations between polymorphisms of rs1364422 and rs429358 by adjusting the uses of these two lifestyle habits. The results showed polymorphism rs1364422 has significant effect on low-HDL-C in females, and polymorphism rs429358 showed significant effect in males.

**Table 5 -:** ORs of relating to low-HDL-C for polymorphisms rs1364422 and rs429358 after adjusting tobacco smoking and alcohol consumption.

SNP	Males (n=20763)		Females (n=20763)	
	aOR (95% CI)	-log(*p-*value)	aOR (95% CI)	-log(*p-*value)
Tobacco smoking				
Yes	1.87 (1.74-2.01)	65.61	1.43 (1.21-1.70)	4.40
No	1.0		1.0	
Alcohol consumption				
Yes	0.70 (0.64-0.77)	12.19	0.51 (0.39-0.67)	5.98
No	1.0		1.0	
rs1364422				
TT	0.94(0.86-1.03)	0.74	1.31(1.21-1.43)	9.35
CT	1.02(0.95-1.10)	0.28	1.13(1.05-1.21)	3.33
CC	1.0		1.0	
rs429358				
GG	1.50(1.03-2.19)	1.46	1.45(1.01-2.10)	1.33
GA	1.36(1.26-1.48)	12.93	1.20(1.11-1.30)	5.07
AA	1.0		1.0	

aOR (95% CI): The odds ratios and 95% confidence intervals of relating to low-HDL-C were estimated for tobacco smoking and alcohol consumption after adjusting age; and estimated for rs1364422 and rs429358 after adjusting age, smoking and alcohol consumption.

## Discussion

The development of low HDL-C could be characterized by genetic, environmental factors, gene-lifestyle interactions (Williams, 2021) as well as gender heterogeneity. We performed a GWAS study to reveal the genetic variants associated with low-HDL-C and found twelve genes related to HDL-C in both genders. Our results also demonstrated that the gene *KLF14* was dominantly related to HDL-C in females, and that the genes *APOC1*, *APOE*, *PVRL2*, and *TOMM40* were significantly associated with low-HDL-C only in males. With reference to the literature, we summarize the gene functions of those with the sex-specific effects in a table (Table S3), and found that the five genes, namely, *KLF14, APOE, TOMM40, APOC1, PVRL2* are found to have associated impact on HDL-C, LDL-C and total cholesterol levels. The KLF family is identified as a class of evolutionarily conserved transcription factors that have a zinc finger domain. It was commonly agreed that these genes regulate many types of cellular processes such as apoptosis, metabolism, proliferation, and differentiation (Lomberk and Urrutia, 2005). Some GWAS results also disclosed that genetic variants surrounding the *KLF14* gene are closely linked to type 2 diabetes, HDL-C levels, metabolic syndrome, HbA1C, and atherosclerosis (Teslovich et al., 2010; Small et al., 2011; Chen et al., 2012; Elouej et al., 2016; Shahvazian et al., 2021). Recently discovered as belonging to the KLF family, *KLF14* was found to regulate cholesterol efflux through inhibiting inflammatory response in macrophages and the expression of *ABCA1* (Wang et al., 2021).

In addition, gender discrepancy is an important issue in how *KLF14* functions. Scholars have examined *KLF14* physiologically with a whole-body knockout mouse model (Argmann et al., 2017). Exploring this transcription factor’s metabolic role on the function of HDL-C and insulin resistance in female mice was positive, but the results showed that the metabolic phenotypes of male mice were not affected by *KLF14* (Argmann *et al.*, 2017). Furthermore, applying a sex-stratified meta-analysis of GWAS data, Small *et al*. discovered that females had larger effect sizes and higher *KLF14* expression than males on the metabolic components, such as HDL-C, triglycerides, BMI, fasting glucose, and fasting insulin (Small *et al.*, 2018). All the aforementioned findings as well as our result suggested that *KLF14* is a female-specific gene for low-HDL-C.

To investigate whether there is a different transcription factor binding site between the two alleles of rs1364422 (allele T and C; *KLF14* gene), PROMO3.0 was applied to find the transcription factor binding sites in DNA sequences (Messeguer et al., 2002). Using the strictest criteria (0%, maximum matrix dissimilarity rate), we found a Yin-Yang member 1 (YY1) binding site in T allele, but not in C allele. As known, “Yin” means negative or repressing; “Yang” means positive or activating. The YY1 is an important transcription factor, included in the GLI-Kruppel class zinc finger proteins. Zinc finger protein can activate and repress a various number of promoters. Weintraub et al. (2017) reported that through promoting DNA interactions and forming dimers, YY1 could promote enhancer-promoter chromatin loops. Hence, its dysregulation would disrupt enhancer-promoter loops and gene expression (Gabriele et al., 2017). From the above, higher frequency of low-HDL-C risk and lower HDL-C levels associated with the T allele of rs1364422 could be realized by creating the YY1 binding site of T allele, but not C allele. 

All four genes related to low-HDL-C in males are located closely in chromosome 19. The genes located in a close region show a higher degree of LD between them, including genes *APOE*, *APOC1*, *PVRL2*, and *TOMM40*. Especially, for the *APOE* gene, Braeckman et al. (1996) revealed that in comparison with the most commonly detected *APOE* ε3/ε3 phenotype, the ε2 allele was linked to a higher HDL-C levels independently of lifestyle factors, *APOE* levels, and age. Frikke-Schmidt et al. (2000) found the variations in *APOE* genotype predicted stepwise decreases with the presence of ε4 in HDL-C for women, but not for men. The data explained two things: firstly, in addition to the well-known increasing effect on non-HDL cholesterol, through a decreasing effect on HDL-C, the ε4 allele could further predispose to coronary heart disease; secondly, through both an increasing effect on HDL-C and a decreasing effect on non-HDL-C, the ε2 allele could exert a protective influence (Braeckman *et al.*, 1996).

For some of the genes identified as related to low-HDL-C in our study, their relationships to metabolic syndrome, HDL-C, or lipid dysfunction have been previously explored in the literature. For instance, *ZPR1* (Galcheva-Gargova et al., 1998; Corton et al., 2000) and *BUD13* (Brooks et al., 2009) were well-documented for lipid level, *HERPUD1* was found related to HDL-C in the Korean population (Oh et al., 2020), and the *APOA5* and *CETP* also showed evidence of contributing to triglycerides, metabolic syndrome, and HDL-C (Talmud et al., 2009; Lin et al., 2016). The rate-limiting step of HDL-C biogenesis was mediated by ABCA1 by transporting cellular excess free phospholipids and cholesterol to an apolipoprotein acceptor (Brunham et al., 2007; Babashamsi et al., 2019). Some studies also provided a role of *ABCA1* between the decreased HDL-C and the increased triglycerides levels (Chung et al., 2010; Liu et al., 2017). Also, recent research illustrated ABCA1’s role in additional metabolic characteristics, revealing its relationship to decreased insulin secretion, sensitivity, and body weight, but to increased blood glucose levels (Brunham *et al.*, 2007; de Haan et al., 2014; Sanchez-Aguilera et al., 2018).

Three genes in the lipase family --*LPL*, *LIPC* and *LIPG*-- were discovered to be related to low-HDL-C in our study*. LPL* is significant in the hydrolysis of core triglycerides of circulating very low-density lipids and chylomicrons (Goldberg, 1996), and its methylation might be involved in triglyceride metabolism and affected by the degree of metabolic disturbances (Castellano-Castillo et al., 2018). Hepatic lipase is synthesized and secreted mainly from the liver (encoded by *LIPC*); it is a glycoprotein in the triacylglycerol lipase family and participates in the hydrolysis of triglycerides and phospholipids (Annema and Tietge, 2011; Kobayashi et al., 2015). Liao et al. (2021) disclosed that HDL-C notably relates to variations in *LIPC* through a genome-wide association analysis. Their study also explored the suppressive role of *LIPC* for triglycerides and found that HDL-C levels might be reduced by a functional haplotype of *LIPC* (Liao *et al.*, 2021). Gene *LIPG* (lipase G) encodes the protein of endothelial lipase, functional on the main substrate, phospholipids in HDL-C, and hydrolysis of phospholipids. West et al. (1996) found that the *LIPG* genetic variant was significantly related to the mean plasma levels of HDL-C. Furthermore, a strong link of the variant and the ratio of HDL-C to LDL-C was apparent in different populations (Ma et al., 2003; Yang et al., 2019). Edmondson et al. (2009) established that increased HDL-C levels could be caused by loss-of-function mutations in *LIPG*. An animal study showed that overexpress of endothelial lipase had decreased HDL-C and lacking endothelial lipase had elevated levels of HDL-C (Cilingiroglu and Ballantyne, 2004).

In conclusion, extending beyond existing studies, we exclusively found that polymorphisms within *ANGPTL8*, *DOCK6,* and *ALDH1A2* were linked to lower HDL-C levels for both genders. Moreover, we demonstrated the low-HDL-C is a sex-specific phenotype, proving that the genes *APOE* and *KLF14* are specific for men and women, respectively.

## Supplementary material

Table S1 - Information of genetic variants relating to low-HDL-C in males.

Table S2 - Information of genetic variants relating to low-HDL-C in females.

Table S3 - Gene functions which are sex-specifically associated significantly with HDL-C reviewed from the literature.
