# Supplementary material for: APOE and KLF14 genetic variants are sex-specific for low high-density lipoprotein cholesterol identified by a genome-wide association study
Source: Genet Mol Biol. 2022 Feb 21;45(1):e20210280. doi: 10.1590/1678-4685-GMB-2021-0280 (PMC8892272; doi:10.1590/1678-4685-GMB-2021-0280)
Supplement: Table S1 - [file 1415-4757-GMB-45-1-e20210280-s1.pdf]

Genetics and Molecular Biology

https://doi.org/10.1590/1678-4685-GMB-2021-0280

Supplementary Material to "*APOE* and *KLF14* genetic variants are sex-specific for low high-density lipoprotein cholesterol identified by a genome-wide association study"

Table S1 - Information of genetic variants relating to low-HDL-C in males.

No.	SNP	chisq.	p-value	chr.	position	ref.	alt.	gene
1	rs28834434	66.62	3.42E-15	8	19917379	C	A	LPL
2	rs2083636	71.95	2.38E-16	8	20007752	T	G	LPL
3	rs35237252	69.1	9.90E-16	8	20012760	C	A	LPL
4	rs1059611	73.09	1.35E-16	8	19967052	T	C	LPL
5	rs10105606	77.29	1.64E-17	8	19970337	C	A	LPL
6	rs10096633	70.57	4.74E-16	8	19973410	C	T	LPL
7	rs10503669	70.76	4.32E-16	8	19990179	C	A	LPL
8	rs12678919	70.54	4.82E-16	8	19986711	A	G	LPL
9	rs13702	73.11	1.33E-16	8	19966981	T	C	LPL
10	rs15285	76.39	2.59E-17	8	19967156	C	T	LPL
11	rs17482753	66.91	2.95E-15	8	19975135	G	T	LPL
12	rs17091905	71.54	2.92E-16	8	19992246	G	A	LPL
13	rs264	39.73	2.35E-09	8	19955669	G	A	LPL

No.	SNP	chisq.	p-value	chr.	position	ref.	alt.	gene
14	rs295	77.55	1.45E-17	8	19958727	A	C	LPL
15	rs2197089	52.29	4.41E-12	8	19968862	G	A	LPL
16	rs2083637	73.72	9.79E-17	8	20007664	A	G	LPL
17	rs320	79.53	5.37E-18	8	19961566	T	G	LPL
18	rs325	71.61	2.82E-16	8	19961817	T	C	LPL
19	rs326	79.77	4.76E-18	8	19961928	A	G	LPL
20	rs328	71.11	3.61E-16	8	19962213	C	G	LPL
21	rs331	75.63	3.77E-17	8	19962894	G	A	LPL
22	rs7016880	52.43	4.11E-12	8	20019235	G	C	LPL
23	rs7816032	69.63	7.60E-16	8	19929380	C	T	LPL
24	rs7841189	70.22	5.64E-16	8	19987865	C	T	LPL
25	rs79236614	72.89	1.49E-16	8	20002949	C	G	LPL
26	rs9644568	44.53	2.14E-10	8	20071071	G	A	LPL
27	rs1883025	39.49	2.66E-09	9	104902020	C	T	ABCA1
28	rs2575876	41.45	1.00E-09	9	104903458	G	A	ABCA1
29	rs2075291	243.82	1.14E-53	11	116790676	C	A	APOA5
30	rs2266788	96.35	1.20E-21	11	116789970	G	A	APOA5
31	rs11216126	62.31	2.95E-14	11	116746524	A	C	BUD13
32	rs1558861	83.16	8.74E-19	11	116736721	C	T	BUD13
33	rs180326	92.47	8.30E-21	11	116753987	G	T	BUD13
34	rs2367970	65.8	5.16E-15	11	116710925	G	A	BUD13

No.	SNP	chisq.	p-value	chr.	position	ref.	alt.	gene
35	rs3825041	94.56	2.93E-21	11	116760991	T	C	BUD13
36	rs7350481	131.15	3.32E-29	11	116715567	T	C	BUD13
37	rs9326246	85.08	3.36E-19	11	116741017	C	G	BUD13
38	rs2160669	100.14	1.80E-22	11	116776891	C	T	ZPR1
39	rs2075290	82.51	1.21E-18	11	116782580	C	T	ZPR1
40	rs3741298	49.74	1.58E-11	11	116786845	C	T	ZPR1
41	rs964184	96.44	1.14E-21	11	116778201	G	C	ZPR1
42	rs1532085	70.87	4.08E-16	15	58391167	A	G	ALDH1A2
43	rs261290	54.15	1.75E-12	15	58386521	T	C	ALDH1A2
44	rs261291	76	3.14E-17	15	58387979	T	C	ALDH1A2
45	rs2043085	73.13	1.32E-16	15	58388755	T	C	ALDH1A2
46	rs1077835	62.45	2.75E-14	15	58431227	A	G	LIPC
47	rs1077834	63.73	1.45E-14	15	58431280	T	C	LIPC
48	rs1800588	64.33	1.07E-14	15	58431476	C	T	LIPC
49	rs2070895	67.12	2.66E-15	15	58431740	G	A	LIPC
50	rs261334	60.06	9.08E-14	15	58434545	G	C	LIPC
51	rs588136	60.92	5.91E-14	15	58438299	C	T	LIPC
52	rs1800774	116.21	5.82E-26	16	56981633	C	T	CETP
53	rs12708980	114.98	1.08E-25	16	56978467	T	G	CETP
54	rs11508026	169.7	1.41E-37	16	56965416	C	T	CETP
55	rs11076175	127.88	1.71E-28	16	56972466	A	G	CETP

No.	SNP	chisq.	p-value	chr.	position	ref.	alt.	gene
56	rs17231506	295.8	5.85E-65	16	56960616	C	T	CETP
57	rs1800775	96.85	9.31E-22	16	56961324	C	A	CETP
58	rs1864163	135.12	4.55E-30	16	56963321	G	A	CETP
59	rs289715	40.79	1.39E-09	16	56974596	A	T	CETP
60	rs2033254	118.87	1.54E-26	16	56976073	T	C	CETP
61	rs3764261	288.93	1.82E-63	16	56959412	C	A	CETP
62	rs4783961	148.31	6.25E-33	16	56960982	G	A	CETP
63	rs708272	187.57	1.86E-41	16	56962376	G	A	CETP
64	rs711752	187.39	2.03E-41	16	56962299	G	A	CETP
65	rs7499892	169.08	1.93E-37	16	56972678	C	T	CETP
66	rs9939224	142.48	1.15E-31	16	56968820	T	G	CETP
67	rs173539	128	1.60E-28	16	56954132	C	T	HERPUD1
68	rs247616	289.85	1.15E-63	16	56955678	C	T	HERPUD1
69	rs247617	286.21	7.08E-63	16	56956804	C	A	HERPUD1
70	rs2156552	54.64	1.36E-12	18	49655298	A	T	LIPG
71	rs3786247	52.71	3.58E-12	18	49592553	T	G	LIPG
72	rs4939883	41.45	1.00E-09	18	49640844	T	C	LIPG
73	rs9958734	51.78	5.71E-12	18	49592028	T	C	LIPG
74	rs2278426	46.14	9.57E-11	19	11239812	C	T	ANGPTL8
75	rs12721046	45.28	1.47E-10	19	44917997	G	A	APOC1
76	rs4420638	40.98	1.26E-09	19	44919689	A	G	APOC1

No.	SNP	chisq.	p-value	chr.	position	ref.	alt.	gene
77	rs429358	55.79	7.67E-13	19	44908684	T	C	APOE
78	rs769449	51.06	8.18E-12	19	44906745	G	A	APOE
79	rs17699030	38.25	4.94E-09	19	11220266	A	G	DOCK6
80	rs3760782	45.93	1.06E-10	19	11235874	C	T	DOCK6
81	rs4804155	40.74	1.42E-09	19	11223619	C	G	DOCK6
82	rs737337	47.01	6.19E-11	19	11236817	T	C	DOCK6
83	rs34342646	40.44	1.66E-09	19	44884873	G	A	PVRL2
84	rs6857	37.4	7.56E-09	19	44888997	C	T	PVRL2
85	rs34404554	39.19	3.09E-09	19	44892652	C	G	TOMM40
86	rs71352238	39.97	2.09E-09	19	44891079	T	C	TOMM40

chisq: The chi-square value; chr: chromosome; ref: reference allele; alt: alternative allele.
