# Supplementary material for: APOE and KLF14 genetic variants are sex-specific for low high-density lipoprotein cholesterol identified by a genome-wide association study
Source: Genet Mol Biol. 2022 Feb 21;45(1):e20210280. doi: 10.1590/1678-4685-GMB-2021-0280 (PMC8892272; doi:10.1590/1678-4685-GMB-2021-0280)
Supplement: Table S2 - [file 1415-4757-GMB-45-1-e20210280-s2.pdf]

Genetics and Molecular Biology

https://doi.org/10.1590/1678-4685-GMB-2021-0280

Supplementary Material to "*APOE* and *KLF14* genetic variants are sex-specific for low high-density lipoprotein cholesterol identified by a genome-wide association study"

Table S2 - Information of genetic variants relating to low-HDL-C in females.

No.	SNP	chisq.	p-values	chr.	position	ref.	alt.	gene
1	rs1364422	37.65	6.66E-09	7	130761222	C	T	KLF14
2	rs2083636	66.59	3.47E-15	8	20007752	T	G	LPL
3	rs35237252	66.61	3.44E-15	8	20012760	C	A	LPL
4	rs1059611	69.99	6.34E-16	8	19967052	T	C	LPL
5	rs10105606	66.81	3.11E-15	8	19970337	C	A	LPL
6	rs10096633	70.12	5.93E-16	8	19973410	C	T	LPL
7	rs10503669	72.45	1.86E-16	8	19990179	C	A	LPL
8	rs12678919	72.52	1.79E-16	8	19986711	A	G	LPL
9	rs13702	64.81	8.44E-15	8	19966981	T	C	LPL
10	rs15285	66.72	3.26E-15	8	19967156	C	T	LPL
11	rs17482753	73.85	9.19E-17	8	19975135	G	T	LPL
12	rs17091905	73.37	1.17E-16	8	19992246	G	A	LPL
13	rs295	66.76	3.19E-15	8	19958727	A	C	LPL
14	rs2083637	68.26	1.51E-15	8	20007664	A	G	LPL
15	rs320	66.45	3.72E-15	8	19961566	T	G	LPL
16	rs325	70.1	6.00E-16	8	19961817	T	C	LPL
17	rs326	64.25	1.12E-14	8	19961928	A	G	LPL
18	rs328	70.52	4.86E-16	8	19962213	C	G	LPL
19	rs331	67.04	2.77E-15	8	19962894	G	A	LPL
20	rs4244457	40.78	1.40E-09	8	20041535	C	T	LPL
21	rs7016880	65.75	5.29E-15	8	20019235	G	C	LPL
22	rs7841189	71.44	3.08E-16	8	19987865	C	T	LPL
23	rs79236614	71.56	2.89E-16	8	20002949	C	G	LPL
24	rs9644568	47.88	4.02E-11	8	20071071	G	A	LPL
25	rs1883025	40.88	1.33E-09	9	104902020	C	T	ABCA1

No.	SNP	chisq.	p-values	chr.	position	ref.	alt.	gene
26	rs2575876	44.5	2.18E-10	9	104903458	G	A	ABCA1
27	rs2075291	233.91	1.61E-51	11	116790676	C	A	APOA5
28	rs2266788	50.88	8.93E-12	11	116789970	G	A	APOA5
29	rs1558861	48.89	2.42E-11	11	116736721	C	T	BUD13
30	rs180326	45.56	1.28E-10	11	116753987	G	T	BUD13
31	rs2367970	39.48	2.68E-09	11	116710925	G	A	BUD13
32	rs3825041	47.75	4.27E-11	11	116760991	T	C	BUD13
33	rs7350481	100.53	1.48E-22	11	116715567	T	C	BUD13
34	rs9326246	49.21	2.06E-11	11	116741017	C	G	BUD13
35	rs2160669	47.1	5.93E-11	11	116776891	C	T	ZPR1
36	rs964184	47.9	3.96E-11	11	116778201	G	C	ZPR1
37	rs10468017	38.64	4.07E-09	15	58386313	C	T	ALDH1A2
38	rs1532085	42.83	5.00E-10	15	58391167	A	G	ALDH1A2
39	rs261290	43.79	3.09E-10	15	58386521	T	C	ALDH1A2
40	rs261291	50.24	1.23E-11	15	58387979	T	C	ALDH1A2
41	rs2043085	40.44	1.66E-09	15	58388755	T	C	ALDH1A2
42	rs4775041	38.21	5.05E-09	15	58382496	G	C	ALDH1A2
43	rs1077835	51.35	7.09E-12	15	58431227	A	G	LIPC
44	rs1077834	53.4	2.53E-12	15	58431280	T	C	LIPC
45	rs117579778	41.18	1.14E-09	15	58421680	G	T	LIPC
46	rs1800588	51.1	8.02E-12	15	58431476	C	T	LIPC
47	rs2070895	52.65	3.69E-12	15	58431740	G	A	LIPC
48	rs261334	62.8	2.31E-14	15	58434545	G	C	LIPC
49	rs588136	61.95	3.53E-14	15	58438299	C	T	LIPC
50	rs12708980	87.34	1.08E-19	16	56978467	T	G	CETP
51	rs11508026	120.88	5.63E-27	16	56965416	C	T	CETP
52	rs11076175	87.26	1.13E-19	16	56972466	A	G	CETP
53	rs1532624	127.06	2.57E-28	16	56971567	C	A	CETP
54	rs17231506	224.41	1.86E-49	16	56960616	C	T	CETP
55	rs1800775	75.89	3.32E-17	16	56961324	C	A	CETP
56	rs1864163	91.13	1.63E-20	16	56963321	G	A	CETP
57	rs2033254	81.01	2.57E-18	16	56976073	T	C	CETP
58	rs3764261	220.97	1.04E-48	16	56959412	C	A	CETP
59	rs4783961	124.08	1.14E-27	16	56960982	G	A	CETP
60	rs6499862	40.66	1.48E-09	16	56957612	G	A	CETP
61	rs708272	141.18	2.20E-31	16	56962376	G	A	CETP
62	rs711752	142.07	1.42E-31	16	56962299	G	A	CETP
63	rs7499892	114.47	1.39E-25	16	56972678	C	T	CETP
64	rs9939224	88.76	5.32E-20	16	56968820	T	G	CETP

No.	SNP	chisq.	p-values	chr.	position	ref.	alt.	gene
65	rs173539	105.27	1.39E-23	16	56954132	C	T	HERPUD1
66	rs247616	219.38	2.30E-48	16	56955678	C	T	HERPUD1
67	rs247617	211.56	1.15E-46	16	56956804	C	A	HERPUD1
68	rs3786247	47.53	4.77E-11	18	49592553	T	G	LIPG
69	rs9958734	47.76	4.27E-11	18	49592028	T	C	LIPG
70	rs2278426	77.34	1.60E-17	19	11239812	C	T	ANGPTL8
71	rs12979813	37.47	7.31E-09	19	11232027	A	G	DOCK6
72	rs3760782	73.93	8.85E-17	19	11235874	C	T	DOCK6
73	rs4804155	67.2	2.56E-15	19	11223619	C	G	DOCK6
74	rs737337	72.99	1.41E-16	19	11236817	T	C	DOCK6

chisq: The chi-square value; chr: chromosome; ref: reference allele; alt: alternative allele.
