# Supplementary material for: APOE and KLF14 genetic variants are sex-specific for low high-density lipoprotein cholesterol identified by a genome-wide association study
Source: Genet Mol Biol. 2022 Feb 21;45(1):e20210280. doi: 10.1590/1678-4685-GMB-2021-0280 (PMC8892272; doi:10.1590/1678-4685-GMB-2021-0280)
Supplement: Table S3 - [file 1415-4757-GMB-45-1-e20210280-s3.pdf]

Genetics and Molecular Biology

https://doi.org/10.1590/1678-4685-GMB-2021-0280

Supplementary Material to "*APOE* and *KLF14* genetic variants are sex-specific for low high-density lipoprotein cholesterol identified by a genome-wide association study"

Table S3 - Gene functions which are sex-specifically associated significantly with HDL-C reviewed from the literature.

Gene	Function
KLF14	1. linked to type 2 diabetes, HDL-C levels, metabolic syndrome, HbA1C and atherosclerosis (Teslovich *et al.*, 2010; Small *et al.*, 2011; Chen *et al.*, 2012; Elouej *et al.*, 2016; Chen *et al.*, 2020; Shahvazian *et al.*, 2021).<br>2. *KLF14* regulates cholesterol efflux through controlling inhibits inflammatory response in macrophages and the expression of *ABCA1* (Wang *et al.*, 2021).
APOE	1. *APOE* plays interactive effects of diet, alcohol, physical activity, adiposity, and smoking with genetic variants of APOE to interpret HDL levels(Braeckman *et al.*, 1996; Williams, 2021).<br>2. It plays an important role in the clearance of cholesterol from circulation (Minihane *et al.*, 2007), and the alleles ε2 and ε3 showed a significant association with the total cholesterol and LDL levels (Seo *et al.*, 2021).<br>3. It acts as the major cholesterol carrier in blood and brain, which also has the highest risk of developing coronary heart disease and Alzheimer's disease(Yousuf and Iqbal, 2015; Lanfranco *et al.*, 2020).
APOC1	1. *ApoC1* participates in lipid transport and metabolism and showed a linkage disequilibrium with APOE gene (Fuior and Gafencu, 2019).<br>2. *ApoC1* is the only known endogenous inhibitor of cholesteryl ester transfer protein (Gautier *et al.*, 2000), and this constitutive action of apoC1 is impaired in coronary artery disease of dyslipidemic patients (Pillois *et al.*, 2012).<br>3. *ApoC1* genetic variants provide evidence for implication in diabetic nephropathy(Tziastoudi *et al.*, 2020) and late-onset Alzheimer's disease (Ki *et al.*, 2002).
PVRL2	1. rs6859 of *PVRL2* was associated with HDL-C, LDL-C and total cholesterol (Mo *et al.*, 2019).<br>2. Genetic variants on chromosome 19 that contains the *APOE, TOMM40, APOC1, PVRL2* genes show a statistically-significant conditional association with cognitive impairment for three secondary phenotypes, CRP, LDL and total cholesterol (Lutz *et al.*, 2019).

Gene	Function
	3. Genetic variants in *PVRL2-TOMM40-APOE* region are associated with human longevity in a Han Chinese population (Lu *et al.*, 2014).
TOMM40	1. *TOMM40* loci are associated with increased low-density lipoprotein-cholesterol (Radovica *et al.*, 2014).
	2. *TOMM40* was associated with the low-density lipoprotein cholesterol level in Chinese patients with type 2 diabetes (Kong *et al.*, 2015).
	3. *TOMM40* may be susceptibility loci for hyper- LDL -cholesterolemia in Japanese individuals (Abe *et al.*, 2015).
